# Parenting Stress Undermines Mother-Child Brain-to-Brain Synchrony: A Hyperscanning Study

**DOI:** 10.1038/s41598-019-47810-4

**Published:** 2019-08-06

**Authors:** A. Azhari, W. Q. Leck, G. Gabrieli, A. Bizzego, P. Rigo, P. Setoh, M. H. Bornstein, G. Esposito

**Affiliations:** 1 Psychology Program, School of Social Sciences, Nanyang Technological University, Singapore, Singapore; 2 Department of Psychology and Cognitive Science, University of Trento, Rovereto, Italy; 3 University of Padova, Padova, Italy; 4 National Institute of Child Health and Human Development, Bethesda, USA; 5 Institute for Fiscal Studies, London, United Kingdom

**Keywords:** Social neuroscience, Cooperation, Human behaviour

## Abstract

Synchrony refers to the coordinated interplay of behavioural and physiological signals that reflect the bi-directional attunement of one partner to the other’s psychophysiological, cognitive, emotional, and behavioral state. In mother-child relationships, a synchronous pattern of interaction indicates parental sensitivity. Parenting stress has been shown to undermine mother-child behavioural synchrony. However, it has yet to be discerned whether parenting stress affects brain-to-brain synchrony during everyday joint activities. Here, we show that greater parenting stress is associated with less brain-to-brain synchrony in the medial left cluster of the prefrontal cortex when mother and child engage in a typical dyadic task of watching animation videos together. This brain region overlaps with the inferior frontal gyrus, the frontal eye field, and the dorsolateral prefrontal cortex, which are implicated in inference of mental states and social cognition. Our result demonstrates the adverse effect of parenting stress on mother-child attunement that is evident at a brain-to-brain level. Mother-child brain-to-brain asynchrony may underlie the robust association between parenting stress and poor dyadic co-regulation. We anticipate our study to form the foundation for future investigations into mechanisms by which parenting stress impairs the mother-child relationship.

## Introduction

A majority of parents will attest to the joys that caregiving a child brings^1^, yet many will also aver that stress is invariably bundled into the parenting experience. Parenting stress arises when a parent’s available coping resources are perceived to be insufficient to meet the demands of parenthood^2^. In consequence, aversive psychophysiological responses towards the parenting role develop^3^. Excessive parenting stress impedes maternal sensitivity^4–6^, incites punitive parental reactions^6^, and adversely shapes the parent-child relationship^6^. Furthermore, the effects of these early impairments are regrettably enduring, predicting poor attachment security^7^ and enhanced externalising behavioural problems in offspring^8–10^.

Synchrony, the temporal coordination of discrete micro-level signals between dyadic partners^11^, is the process by which the physiology and behaviour of mother and child are coordinated into a selective affiliative bond that matures into an enduring attachment^12^. Synchrony between mothers and their infants has been observed as early as 3-months during face-to-face interactions^13^. A higher incidence of interactional synchrony, marked by shared similar emotions and mutual engagement and turn-taking, is indicative of dyads where partners were sensitively attuned to each other^14^. Mother-child synchrony subserves interpersonal emotional co-regulation which contributes to the child’s emotional self-regulation and adaptive physiological response to social stress^15^.

From a biobehavioural perspective, synchronous dyadic interaction is facilitated by both behavioural reciprocity (e.g., facial expressions, gaze patterns) and physiological oscillators (i.e., biological rhythms)^12^. Although parenting stress diminishes overt reciprocal engagement between parent and child, it has yet to be discerned whether overt reduction in engagement can be traced to an isolatable underlying physiological mechanism that supports dyadic coordination. The present study sought to investigate how brain-to-brain synchrony, indicated by the alignment of two independent signals across time, varies as a function of parenting stress during a passive joint dyadic attention task. Utilising tandem functional Near-infrared Spectroscopy (fNIRS), activation patterns in the prefrontal cortices (PFC) of mother and child were recorded simultaneously while dyads watched a series of animation videos together. The PFC has long been known to play a major role in higher-order cognitive processes^16^. As its heightened activity is also associated with active “top-down” regulation of emotional responses^17^, it is plausible that the PFC is differentially recruited in mothers who report higher as compared to lower parenting stress. Two recent functional Magnetic Resonance Imaging (fMRI) studies showed that parental stress modulated orbitofrontal cortex (OFC) activation when a mother viewed images of her own child^17,18^, reinforcing involvement of the PFC in maternal brain mechanisms related to parenting stress. We embarked on this study with one principal hypothesis, anticipating that mothers who experience greater parenting stress would exhibit less dyadic prefrontal cortical synchrony when engaging in a task with their child.

## Methods

### Participants

A total of 31 mother-child dyads (13 girls, 18 boys) were recruited through online forums and social media groups (Mean age of mothers = 34.9 years, ±4.16 years; Mean age of children = 41.6 months, ±6.1 months). Mothers were required to be above 21 years of age with a biological child between the ages of 36 and 48 months at recruitment. Both mother and child had to be residing in Singapore and must not suffer from severe cognitive deficits, visual or hearing impairments, or major diseases that could prevent them from understanding and responding to the experimental tasks. Informed consent was obtained from all participants (or their mother in case of minors) prior to the start of the study, and each dyad was remunerated on completion of the study. All methods were performed in accordance with the relevant guidelines and regulations and the study was approved by the Institutional Review Board of Nanyang Technological University. All data are available at this URL: 10.21979/N9/CTR0YX.

### Experimental procedure

**Figure 1:**
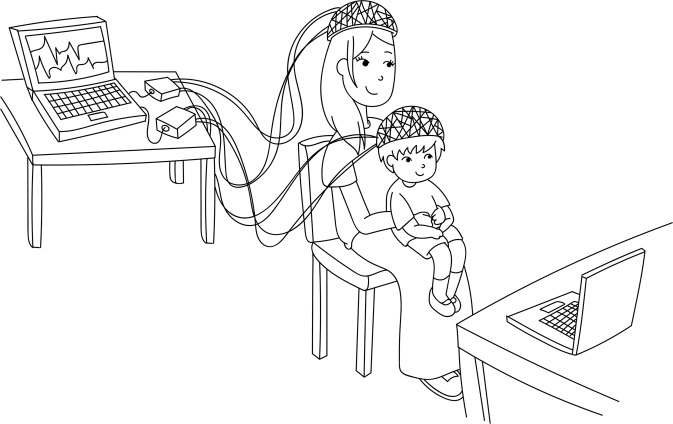
Illustration depicting the set-up of devices and sitting arrangement of mother-child dyads during the experimental sessions. Figure illustrated by Nur Hasyimah Bte Johari.

In the first part of the study, mothers completed home-based online questionnaires which included demographic questions and a 36-item Parenting Stress Index - Short Form (PSI-SF)^19^, used to examine the perceived total stress that a mother has with regard to her parenting. In the second part of the study, each dyad was randomly assigned to one of six video sequences prior to the start of an fNIRS experiment. Upon entering the child-friendly laboratory, a research assistant explained the entire task to the mother, after which the researcher directed the dyad into an experimental room. The mother sat on a chair while her child sat on her lap throughout the experiment (Fig. 1). NIRS caps of appropriate sizes were placed on the heads of mother and child and recording was conducted in a tandem hyperscanning mode. A short 1-min video clip from the movie ‘Moana’ was screened as a distraction while the NIRS devices were set up. After the caps were in place, the optodes were adjusted to optimise signal quality. The video was screened on a laptop that was placed at the centre of the table at a distance of 40 cm from the dyad. Once the video and fNIRS data-recording started, the researchers exited the experimental room. At the end of the session, the researchers re-entered the room and proceeded to help the dyad remove the devices while a video screening of ‘Peppa Pig’ was played to distract the child. Lastly, the mother was debriefed about the study and remunerated.

### Parenting stress index - short form

The Parenting Stress Index - Fourth Edition (PSI-4)^19^ is a screening and diagnostic tool used to measure perceived parenting stress in the parent-child system for a child who is between 1 month and 12 years of age. The short format has 36 items and has been found to have high reliability (alpha coefficient = 0.98)^20^ and validity^21–23^. Parents read the statements and then rate their response on a 5-point Likert-scale, where 1 indicates strongly disagree and 5 indicates strongly agree. The sample for our study had high internal consistency (alpha coefficient = 0.90).

The Parenting Stress Index - Short Form (PSI-SF) is created by extracting items from the original PSI and consists of three main sub-scales. The 12-item Parental Distress subscale measures personal factors of the parent that contribute to stress and consists of items such as the perceived restriction experienced after having a child (e.g. ‘Since having my child I have been unable to try new and different things.’). The 12-item Parent-Child Dysfunctional Interaction subscale measures the extent to which the parent experiences satisfaction from interactions with her child and the extent to which the child meets her expectations. It includes items such as ‘My child is not able to do as much as I expected’. The 12-item Difficult Child subscale provides an indication of the parent’s perceptions of her child’s characteristics and has items such as ‘My child generally wakes up in a bad mood.’). The total stress perceived by the mother is the sum of the scores of the three subscales, with higher scores representing greater parenting stress.

### Video stimulus

**Figure 2:**
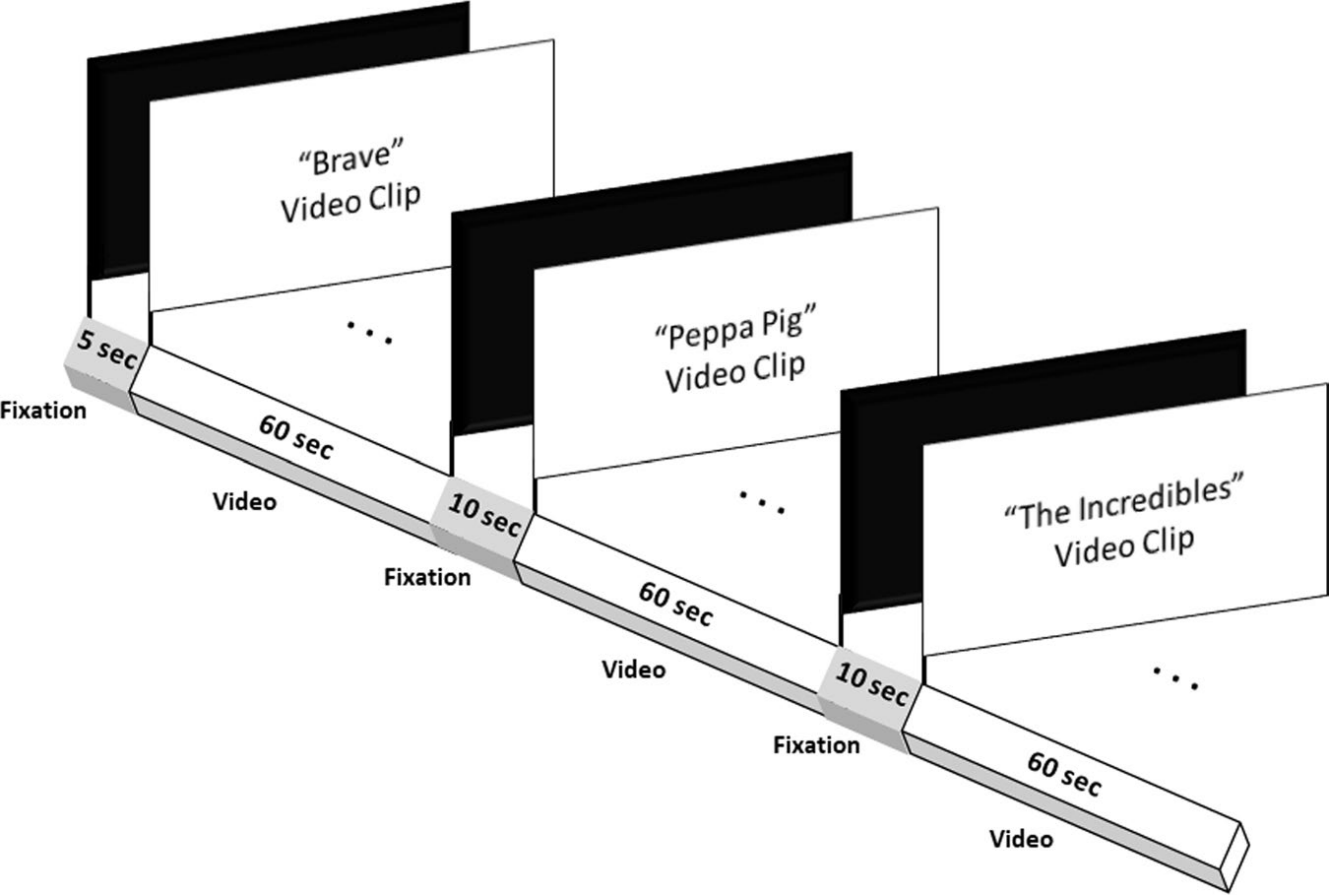
Schematic diagram representing the video stimuli screened to participants. A 5-sec fixation point “+” was presented before the onset of the first video clip. Three 1-min video clips were screened in total, with an inter-stimulus interval of 10 sec between each clip. The order of presentation of the three video clips was randomised such that six video sequences were generated. Mother-child dyads were randomly assigned to one of six video sequences.

This study employed a passive joint video attention task because of the familiarity of this activity for both mothers and children. Three 1-min animation videos from Brave, Peppa Pig and The Incredibles, were selected because of their different emotional valences and audio-visual complexities, so as to increase the generalisability of this dyadic activity. The video clips were edited to equate average volume and brightness. A 5-sec fixation cross was added to the start of each video clip, and a 10-sec inter-stimulus interval (ISI) fixation cross was added between each clip (Fig. 2). To control order effects, the three animation clips were randomised to create a total of six different sequences. The video stimulus was screened on a 15-inch Acer Laptop with both brightness and volume set on the laptop to 60%.

Using Python and the FFmpeg software (v. 3.4.4), each video clip was analysed for its visual complexity (i.e. video complexity) by extracting the video at 12 frames per second (FPS). The audio intensity and audio fundamentals of the video were analysed by first converting the video to an audio file on FFmpeg, before using Praat software (v. 6.0.46) to extract the audio information of the clip. Emotional valence was calculated microanalytically by rating each sec of the video as either positive, neutral, or negative, and the sum of the three 1-min video clips was used as the respective rating of positivity. Results of the visual complexities, audio intensities, audio fundamentals, and valences for each video clip are reported in Table 1.

**Table 1:** Values of video complexity, audio fundamentals (pitch), audio intensity, and valence of the three animation clips: Brave, Peppa Pig, and The Incredibles.

Clip	Visual Complexity (bytes)	Pitch (Hz)	Audio Intensity (W/m^2^)	Video Valency
Brave	658119.31	245.26	59.89	45
Peppa Pig	468369.81	218.37	62.5	59
The Incredibles	423005.66	271.64	56.3	−54

### Functional near-infrared spectroscopy (fNIRS) data acquisition

#### fNIRS experimental set-up and pre-processing

Prefrontal cortical (PFC) activity was measured in tandem mode using the non-invasive fNIRS neuroimaging system (NIRSport, NIRx Medical Technologies LLC). This equipment has a scan rate of 7.81 Hz and employs LED emission with source wavelengths of 760 nm and 850 nm. The mother and child NIRS caps have 8 LED sources and 7 detectors to collect optical signals. The inter-optode distance was kept at the optimal maximum of 3 cm. NIRSlab (NIRS v.205 software) software was used to configure a 20-channels-recording system of the PFC, utilizing an 8x7 source-detector montage. fNIRS allows for monitoring local blood oxygenation, with more active brain areas exhibiting a greater concentration of oxygenated haemoglobin (HbO) relative to reduced haemoglobin (HbR)^24^.

NIRSlab software was used to conduct pre-processing and analysis of NIRS data. First, channels were inspected and those with a significant background noise of gain >8, CV > 7.5 were determined as noisy and excluded from further pre-processing. Next, markers for onset of each of the 3 video stimuli were added. This was followed by manual removal of discontinuities and spike artefacts. A band-pass filter of 0.01–0.2 Hz was applied to remove any physiological and slow signals and baseline shift variations. For each channel, haemodynamic states were then calculated, and the pre-processed signals were converted to changes in concentration of HbO and HbR using the modified Beer-Lambert law. Finally, the signal was manually inspected by two independent coders to detect the presence of further artifacts.

#### General linear model (GLM) analyses

Two levels of analyses were conducted for NIRS data: within-subject analysis (first-level) and group-level analysis (second-level). In the first-level analysis, a haemodynamic response function (HRF) was specified and pre-whitening was omitted. HRF towards each video was plotted against a time axis using a convolution design matrix where each point of the matrix was checked against the order of stimulus presentation the participants received. This was followed with the application of a Discrete Cosine Transformation (DCT) temporal parameter with a high-pass period cut-off of 128 sec. Next, a Gaussian Full Width at Half Maximum (FWHM) 4 model was applied, and for each individual participant General Linear Models (GLMs) were obtained based on the HbO signals. Using the GLMs, beta-coefficients for each of the 3 videos were obtained and then aggregated to obtain an average beta-coefficient. At the second-level analysis, beta-coefficients obtained from HbO of each participant were aggregated into a group-level GLM.

#### Dynamic time warping (DTW) time-series analyses

To quantify synchrony between mother and child, a robust algorithm known as Dynamic Time Warping (DTW) was performed on pre-processed time-series data of each dyad. DTW allows for similar but out-of-phase shapes to match within the same time period^25^, resulting in the transformation of time-series data via arrangement of all sequence points, thereby optimising the alignment of any two sequences^26^. DTW has been previously used in electrocardiogram^27^ and word recognition studies^28–30^. A distance index, also known as a cost function^26^, was generated for each dyad. The greater the distance index, the lesser the synchronisation between members of a dyad^31^.

#### Cluster grouping of channels

**Figure 3:**
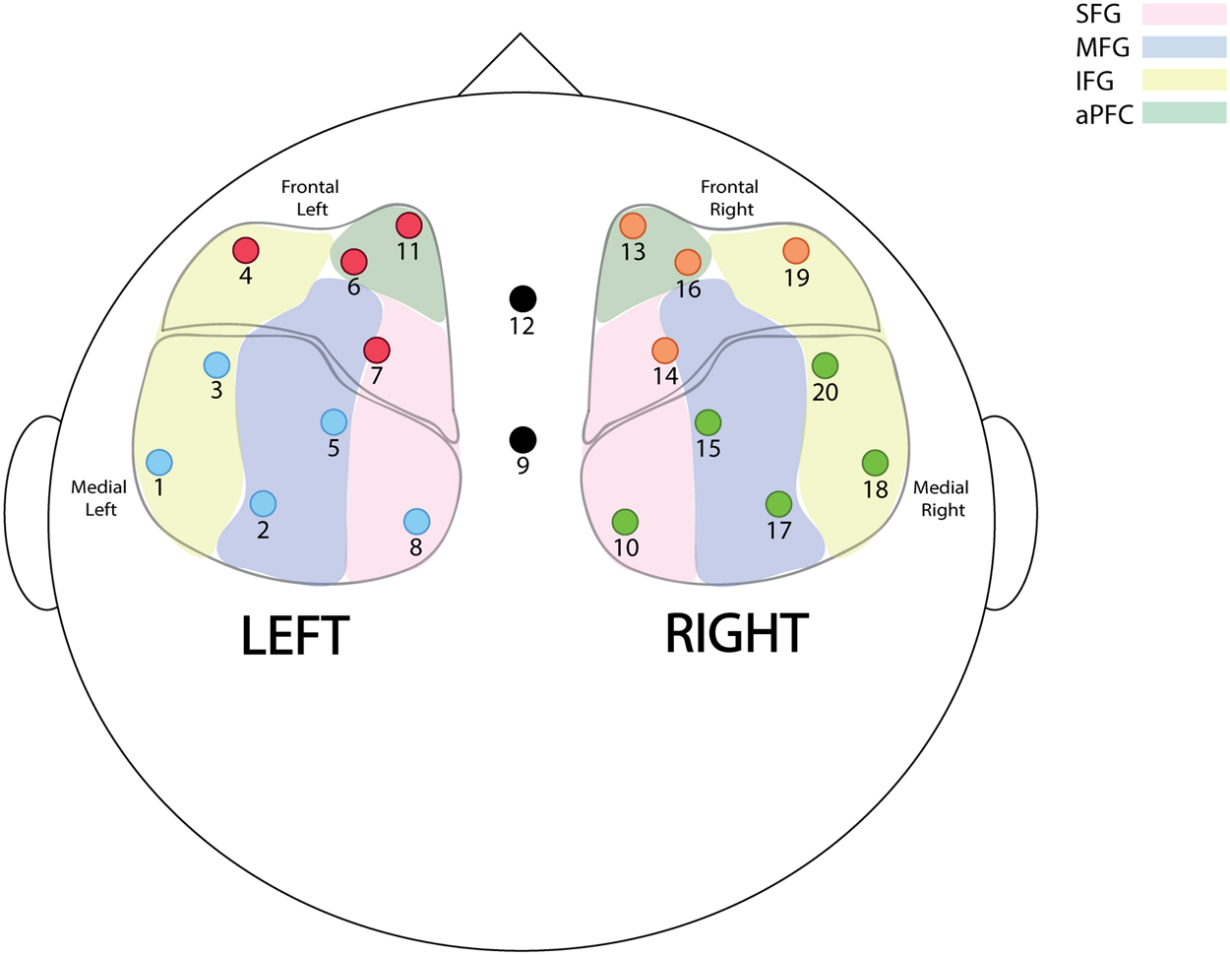
Schematic diagram depicting locations of the 20 optode channels and their corresponding positions with respect to the superior frontal gyrus (SFG), middle frontal gyrus (MFG), inferior frontal gyrus (IFG), and anterior prefrontal cortex (aPFC). Channels were grouped into the following clusters: frontal left, frontal right, medial left, and medial right.

Given that brain regions are interconnected in networks, investigating PFC in clusters instead of single channels provided a more meaningful and realistic interpretation of the results. Channels were clustered into 4 regions of the PFC - frontal left, frontal right, medial left, and medial right. The four clusters were formed by grouping different channels in near proximity. Figure 3 shows the clusters of channels as well as the Brodmann Area implicated in each cluster.

### Analytic plan: general linear model

#### Preliminary analyses

All analyses were done in R studio (version 1.0.153, R-core 3.4.2). For each cluster, beta coefficients of the HbO signal were summed and averaged across the channels within that cluster. Preliminary analyses were conducted where child’s gender, mother’s age, and video valence were fitted into 3 linear models individually as factors against each cluster. Video complexity and audio intensity and fundamentals were included as covariates in all three regression models. The analyses were conducted twice - once for mothers and once for children.

#### Descriptive analyses

The means and standard deviations of the averaged beta values for all 4 clusters for both mothers and children are reported.

#### Inferential analyses

A multivariate linear regression was conducted for each of the 4 clusters, fitting parenting stress as a factor with video complexity and audio fundamentals as controls (i.e., Beta = Parenting Stress + (Video Complexity + Audio Fundamentals)). A significant relation between a cluster and parenting stress implies that, while engaging in a joint task (i.e., watching videos), the level of parenting stress reported by the mother is associated with activation in that particular PFC cluster. False Discovery Rate (FDR) correction was applied to all p-values to correct for multiple channel comparisons. Regression analysis was executed twice - once for mothers and once for children.

### Analytic plan: synchrony analyses

#### Preliminary analysis

Distance indices for each PFC cluster were summed and averaged across all the channels in each cluster, resulting in an overall distance index for each cluster. We employed normalised distance indices, which applied correction according to the length of the signals thereby allowing us to compare signals of different lengths (e.g., when only a portion of the signal is kept for one individual within the dyad while the other individual has the complete set of signals). A preliminary analysis was conducted where gender, maternal age, and video valence were fitted into 3 models individually as factors against each of the four cluster’s distance index. Video complexity and audio fundamentals were included as covariates in all three regression models.

#### Descriptive analyses

The means and standard deviations of the distance indices for all 4 clusters are reported.

#### Inferential analyses

Similarly, general linear regressions were conducted for each of the clusters. A model with parenting stress as a factor with video complexity and audio fundamentals as controls was fitted with each of the 4 clusters’ distance indices (i.e., Distance Index = Parenting Stress + (Video Complexity + Audio Fundamentals). A significant relation between the distance index and parenting stress within a cluster implies that level of synchrony between mother and child for that particular PFC region is associated with the level of parenting stress reported by the mother. FDR correction was then applied to all p-values to correct for multiple comparisons.

## Results

### General linear model

#### Preliminary results

Multiple linear regression analyses were performed for mother’s age, child’s gender, and video positivity, with video complexity and audio fundamentals as covariates, on both mothers and children. For mothers, no cluster survived p-value correction for any of the regression analyses. For children, results of the regression indicated a significant relation in the frontal left PFC for the GLM model with mother’s age as a factor (*R*^2^ = 0.05818, F(3,245) = 5.045, p < 0.01) and child gender as a factor (*R*^2^ = 0.05816, F(3,245) = 5.043, p < 0.01), even after p-value correction (see Table 2). A closer examination of the results indicated that video complexity was the only variable with a significant beta coefficient across all three regressions (see Table 3). Given that video complexity was a variable that was controlled throughout the study, we proceeded to exclude mother’s age and child gender while retaining video complexity as a control in subsequent analyses.

**Table 2:** Beta coefficients of video complexity in the frontal left PFC of children for regression models with mother’s age, child’s gender and video positivity.

Models	*β*	SE	t	p
Mother’s Age	−1.04 · 10^−9^	2.68 · 10^−10^	−3.87	0.000139
Child’s Gender	−1.04 · 10^−9^	2.68 · 10^−10^	−3.87	0.000139
Video Positivity	−1.14 · 10^−9^	3.02 · 10^−10^	−3.79	0.000190

**Table 3:** Beta coefficients of video positivity and video complexity co-variates in the frontal right PFC of children.

Variable	*β*	SE	t	p
Video Positivity	−3.24 · 10^−6^	1.40 · 10^−6^	2.31	0.0214
Video Complexity	−8.88 · 10^−10^	3.49 · 10^−10^	−2.55	0.0114

For children, results of the regression analyses also indicated that video positivity was a significant covariate in both frontal left (*R*^2^ = 0.05816, F (3,246) = 7.595, p < 0.01) and frontal right (*R*^2^ = 0.02988, F (2,259) = 3.988, p < 0.05) clusters of PFC. In the frontal left cluster, the covariate, video complexity, was the only variable with a significant beta coefficient. Given that video complexity was added as a covariate in all regression models, we could exclude video positivity from subsequent analyses in the frontal left cluster. However, in the frontal right PFC, beta coefficients of video positivity were also found to be significant and, thus, included in subsequent analyses with parenting stress only for this cluster.

#### Descriptive results

The means and standard deviations of the beta values for the clusters of both mothers and children are reported in Tables 4 and 5, respectively.

**Table 4:** Means and Standard Deviations of the beta values of the clusters for mothers.

Clusters	Mean beta values	Standard Deviation
Frontal left	−5.25 · 10^−7^	2.11 · 10^−6^
Frontal right	8.82 · 10^−7^	2.92 · 10^−6^
Medial left	1.07 · 10^−6^	6.35 · 10^−6^
Medial right	4.09 · 10^−6^	6.97 · 10^−6^

**Table 5:** Means and Standard Deviations of the beta values of the clusters for children.

Clusters	Mean beta values	Standard Deviation
Frontal left	−2.71 · 10^−6^	2.74 · 10^−6^
Frontal right	−1.02 · 10^−6^	1.22 · 10^−6^
Medial left	−2.79 · 10^−6^	2.62 · 10^−6^
Medial right	−1.55 · 10^−6^	5.00 · 10^−6^

#### Inferential results

Multivariate regression analysis was used to test if parenting stress was significantly associated with cluster activation in the PFC for both mother and child. For mothers, no significant relation was found between parenting stress and PFC cluster activation. For children, after p-value correction, the GLM with parenting stress as a factor was significant in the frontal left cluster (*R*^2^ = 0.05818, F(3, 245) = 0.008308, p < 0.05). However, a closer examination indicated video complexity as the sole variable with a significant beta coefficient (*β* = 31.036e-09, p < 0.01). Given that video complexity was added as a control, we concluded that parenting stress was not significantly related to cluster activation in children.

### Synchrony analyses

#### Preliminary results

Multiple linear regressions reported no significant relations between synchrony in the 4 clusters with mother’s age, child's gender, and video positivity for both mothers and children.

#### Descriptive results

Means and standard deviations of the distance indices for the clusters are reported in Table 6.

**Table 6:** The four prefrontal cortical clusters and their associated channels, along with sample size and demographic information (mother’s age, child’s age, child’s gender).

Cluster	Channels	Brain Areas	Brodmann Areas	N	Mother’s Age	Child’s Age	Child’s Gender (Male, Female)
Frontal Left	4,6,7,11	Anterior PFC, Middle frontal gyrus, Pars orbitalis of Inferior frontal cortex (IFC)	BA47L, BA10L, BA46L	31	35.2 ± 0.3	41.6 ± 6.1	18, 13
Frontal Right	13,14,16,19	Anterior PFC, Middle frontal gyrus, Pars orbitalis of Inferior frontal cortex (IFC)	BA10R, BA46R, BA47R	30	35.2 ± 4.3	41.7 ± 6.1	18, 12
Medial Left	1,2,3,5,8	Dorsolateral PFC (DLPFC), Lateral PFC, Broca’s area of IFC, Lateral DLPFC, Superior frontal gyrus	BA46L, BA45L, BA09L, BA08L	27	35.4 ± 4.5	41.1 ± 5.4	15, 12
Medial Right	10,15,17	DLPFC, Superior frontal gyrus, Lateral DLPFC, Lateral PFC, Intermediate frontal cortex, Pars triangularis of IFC	BA08R, BA09R, BA46R, BA45R	29	35.3 ± 4.3	41.2 ± 5.7	16, 13

#### Inferential results

**Figure 4:**
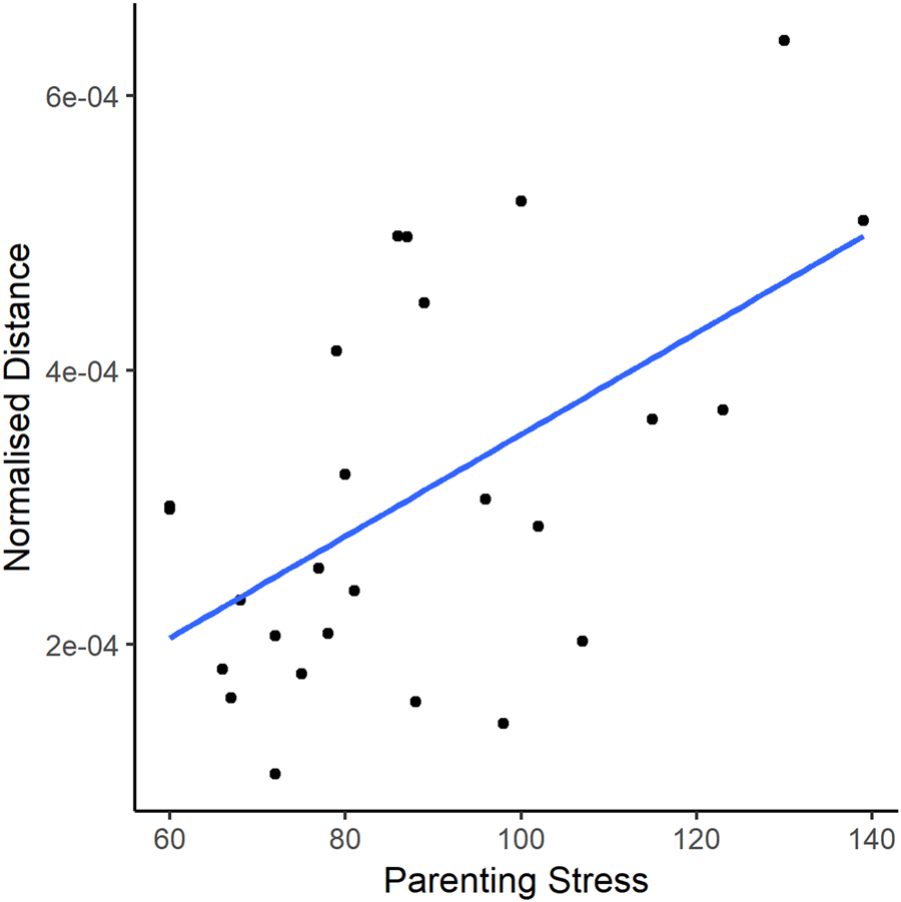
Scatterplot depicting the positive linear relation between reported parenting stress by mothers and normalised distance index in the medial left cluster (r = 0.56, p = 0.0032; FDR corrected).

Multiple linear regression was used to test if parenting stress was significantly associated with PFC synchrony between mother-child dyads in the different clusters. Parenting stress was not related to synchrony in the frontal left (*R*^2^ = 0.01602, F(3, 226) = 1.226, p > 0.05), medial right (*R*^2^ = 0.01664, F(3, 261) = 1.472, p > 0.05), or frontal right (*R*^2^ = 0.03434, F(3, 231) = 2.738, p > 0.05) PFC clusters. However, while holding video complexity and audio fundamentals constant, parenting stress was significantly related to synchrony in the medial left PFC cluster (*R*^2^ = 3.730e-06, F(3,305) = 3.876, p < 0.01). This cluster consists of areas corresponding to the left inferior frontal gyrus (IFG), the frontal eye field (FEF), and the dorsolateral PFC. A follow-up Pearson correlation test was conducted to determine the direction of the relation and, as hypothesised, parenting stress was found to have a significant positive correlation with distance index (r = 0.556, p = 0.0032) (Fig. 4). Given that a larger distance index implies weaker synchrony in the dyad, synchrony in this PFC cluster decreases as parenting stress increases.

## Discussion

Synchrony between mother and child is borne out of a unique dyadic bond that is continuously shaped by reciprocal social interactions. However, the stressors of parenthood may undermine the quality of dyadic engagement, and suboptimal levels of synchrony may impair the development of healthy attachment and emotional regulation in the child. In the present study, we found a significant association between parenting stress and dyadic mother-child inter-brain synchrony within the medial left PFC cluster. Specifically, higher parenting stress is associated with less mother-child synchrony in the left inferior frontal gyrus (IFG), the frontal eye field (FEF), and the dorsolateral PFC, thus partially supporting our hypothesis.

In a recent study by^32^, the authors postulated that higher stress may lead to a mother’s diminished acceptance of her child, thus reducing her flexibility to understand and adaptively respond to her child’s needs. The medial PFC is involved in mental inferencing abilities^33–35^. Within the medial left clusters are Brodmann Areas 8 and 9 which have garnered much attention for their role in Theory of Mind (ToM) processes, implicating the ability to infer the mental states of others^36^. Diminished inter-brain synchrony in these regions indicates a localised dissimilarity between mentalisation processes of mothers with higher levels of parenting stress and their children while engaged in a joint viewing task. Two possible processes may have led to this result. First, similar to^32^, mothers with elevated parenting stress may command comparatively less adaptive flexibility, which suggests that they might not be attuned to their child’s emotional cues, and are less able to adopt the perspective of their child while viewing the videos. Bearing in mind that this joint activity was passive with no face-to-face communication, this finding points to the extent to which mothers were able to instinctively adjust their mentalisation and align it with that of their child, with minimal feedback in the form of behavioural cues. Alternatively, the lack of dyadic similarity in activation patterns involved in mentalisation could be due to reduced ToM capacities in the child, which have been previously reported to be associated with diminished dyadic synchrony^13^. From 3- to 4-years of age, children develop ToM and begin to understand that other individuals may possess beliefs about the world that are distinct from their own^37^. Because parenting stress compromises the quality of maternal-child synchrony, it is possible that children of highly stressed mothers have not developed ToM capacities to empathise with fictional characters screened to them, leading to divergent cognitive responses between mother and child while engaging in the joint viewing task. In all likelihood, both interpretations could contribute to the observed inverse association between parenting stress and mother-child brain-to-brain synchrony.

Having experienced ample social interactions together, our mother-child dyads would have had numerous opportunities to share emotional experiences^38^. Mutual exchange of emotions has been found to lead to emotional coordination during which partners develop similar responses to various emotional situations^39^. Mother-child dyads who engage in more open communication would find it relatively easier to express their feelings to each other^40^, thus facilitating the development of emotional coordination. However, parenting stress impairs the openness of dyadic social exchange which potentially inhibits the formation of parallel attuned emotional responses^40^. This circumstance presents a third plausible process through which parenting stress reduces synchrony within the left medial PFC. Previous studies have documented the role of the medial PFC in emotional regulation^41–43^ and in memory retrieval of event-specific emotional responses^44^. Because mother and child viewed emotionally salient video content together, greater similarity in event-specific responses and regulatory processes could have occurred in dyads where mothers reported less parenting stress, due to the enhanced quality of emotional coordination that these dyads typically experience in daily life. By contrast, dyads with mothers who reported greater parenting stress could have experienced poorer social exchange and emotional coordination, resulting in diminished synchronous activation patterns with their child when emotional responses were elicited.

It is crucial to take the findings of this study with the following limitations in mind. First, maternal parenting stress was measured using a self-report questionnaire and as such was not an objective measure. Social desirability bias could have affected the responses such that mothers answered in a way that allowed them to be viewed favourably by the researchers. Mothers might either exaggerate or under-report their parenting stress levels depending on their own individual perception of their current parenting and family situation. Nonetheless, the measure we used is a self-report of the mothers’ phenomenological experience of their own parenting stress level, and therefore has validity. Second, our experimental design required children to sit on their mothers’ laps so as to reduce the stress experienced by young children in an unfamiliar, albeit child-friendly, laboratory environment. However, physical touch between mother and child could also regulate the intensity of emotional responses, thus affecting the synchrony of dyads. This positioning of mother and child, which would reduce stress, would actually work against the hypothesis, and so our results may be conservative. Nonetheless, future studies may adopt experimental designs that better control factors (e.g., touch) which may contribute to dyadic synchrony. Third, this study was cross-sectional in design, and so direction of effects are undetermined. However, we collected parenting stress data temporally in advance of fNIRS data collection, supporting antecedent directionality. Last, this study focused on a specific region of the brain - the prefrontal cortex – which leads to only a partial understanding of the effects of parenting stress. Obtaining signals from other cortical and subcortical regions of the brain would allow a more comprehensive interpretation of the present findings. Nonethless, we found specificity of effects within different regions of the PFC.

## Conclusion

Although caregiving is a naturally rewarding experience, it presents mothers with stressors of parenthood that need to be adapted to and coped with in everyday life. Parenting stress has been shown to impair mother-child behavioural synchrony, but our study is the first to demonstrate that its debilitating effects can be traced to specific neurophysiological mechanisms. In this study, we learned that parenting stress significantly reduces mother-child inter-brain synchrony in the left medial prefrontal cortex when dyads engage in a typical joint attention task. Having established the deep-rooted association between parenting stress and mother-child brain synchrony, our study delivers the message that a mother’s psychological well-being is fundamental to the quality of a mother-child relationship. Given the importance of mother-child synchrony as a critical co-regulatory process in emotional development, this mechanism may potentially underlie the robust associations between parenting stress and children’s externalising behaviours in later development.
